# Medical News and Bulletin

**Published:** 1843-02-18

**Authors:** 


### MEDICAL NEWS AND BULLETIN.

### MEDICAL LIBEL.

*Beale* v. *Self.*

The plaintiff and defendant in this cause, which [illegible]s tried in the Court of Common Pleas last week, [illegible] both practitioners residing in Stepney.

The defendant had asserted that the plaintiff had [illegible]properly treated one Mrs. Neck, and also that he [illegible]as a dissecting-room beadle.

[illegible]nd first, of the first.

Mr. Beale attended Mrs. Neck, the wife of a schoolmaster, and she died after an illness of nine days, during which the symptoms were somewhat obscure. On a post-mortem examination, it was found that "a portion of the smaller intestines were twisted or strangulated to the extent of about six inches, and this part was approaching to gangrene."

Mr. Self, on the termination of the case, asked the husband if leeches had been used; and on hearing that six had been applied, answered that there ought [illegible] have been at least forty or fifty.

This is the chief part of the evidence regarding Mr. Self's opinion of the treatment of Mrs. Neck; but [illegible] [illegible]elief that Mr. Beale was not a surgeon was re[illegible]ed more than once. Thus it appeared, from the [illegible]ce of Mrs. Burt, that she had formerly employ[illegible]r. Self, but, in compliance with the wish of her [illegible] and friends, had engaged Mr. Beale to attend [illegible] her approaching accouchement. Mr. Self cal[illegible] her, and persuaded her to change her mind, [illegible]g her that there was no such person as Mr. [illegible]mas Beale, a surgeon; that Mr. Beale had been [illegible]eadle to a hospital, and that the office of such a man was to clean away the guts and garbage.

Other witnesses deposed to the anatomical, surgical, and obstetrical studies of the plaintiff, and to the opportunities he had enjoyed of acquiring anatomical knowledge, as dissecting beadle to the London Hospital.

His diploma from the College of Surgeons was also put in.

The jury found a verdict for the plaintiff, damages £100.

In any fatal case, a preferable, or, at least, a different treatment, may afterwards be suggested; and, perhaps, it would have been better to prescribe a greater number of leeches (not forty or fifty, however) in Mrs. Neck's case.

The other part of the business is less doubtful. The man who rises from the lowest drudgery of the profession to be a member of the College of Surgeons, must have some good stuff in him, and need never be ashamed of his elevation. Mr. Self, however, does not appear to have known, till too late, that his rival really *was* a member of the College; so that this lawsuit, like so many others, originated in a misunderstanding.*

Another action for the same libel, brought by the same plaintiff against a different defendant, was compromised by a verdict for the plaintiff, with 40s. damages.—*Lon. Med. Gaz.* Dec. 30, 1842.

### DISCOVERY OF SPERMATOZOA WITHIN THE MAMMIFEROUS OVUM.

We understand that at the meeting of the Royal Society on the 8th instant, a paper by Dr. Martin Barry was read, announcing his discovery of spermatozoa *within the mammiferous ovum.* The ova were those of the rabbit, taken, twenty-four hours *post coitum*, from the Fallopian tube.—*London Lancet*, Dec. 17, 1842.

### NEW PERIODICAL AT MILAN.

A fortnightly medical periodical has been recently established at Milan, (a city of a very poor country in science,) edited by Professor Panizzi and Dr. Bertani, called the "Gazetta Medica," divided into five sections; the first and principal comprising reports of cases in the hospitals of Austrian Italy, the rest being devoted to original medical essays, reviews of works, matters of a speculative nature, bibliographical notices, and advertisements. The proprietors have appealed to the practitioners of the Lombardo-Venetian kingdom for the results of their experience, and to the medical public of Europe, generally, for contributions in the Italian, Latin, German, French, or English languages. A volume and index are to be completed yearly. The price is only about sixpence per number, and much less in proportion to annual subscribers. Among the contents of the first five numbers are papers by Professors Flarer, Frank, and Corneliani, and Drs. Bertani, Casorati, Dubini, Trinchinetti, Giovanni, Verga, A. Bianchi, Fabrizi, Gola, &c. If the editors continue true to their professions, this journal will be a valuable record of the medical news of Northern Italy.—*Ibid*, Dec. 31.

Borden, a successful French physician, who lived in the palmy days of hyperbole, was found dead in his bed, on which catastrophe it was remarked "that Death was so much afraid of him that he could get the better of him only while asleep!"

### NEW LUNATIC ASYLUM AT DIJON.

A general asylum for the insane has just been founded at Dijon, on a magnificent plan, and of a fine architectural design, on the spot formerly occupied by a Carthusian convent. Besides the patients of the department, those of the neighbouring ones will also be received. A chief physician will reside as director in the establishment, which will be capable of containing more than 400 patients. Eleven *hectares* of land are annexed to it, the cultivation of which by the patients, whose situation admits of it, will be one of the most powerful means of cure, a fact demonstrated for some years at Paris, both at Bicétre and the Salpêtrière.—*Lon. Med. Gaz. from Gazette Medicale*, Dec. 3d, 1842.

### THE TITLE OF DOCTOR.

In English law the doctorate or highest degree in the Academies, is, in point of rank, analogous to knighthood, and is classed next to it. Sir Edward Coke says "esquires and gentlemen are only names of worship, not of dignity." And Sir William Blackstone says, in the art. Precedency in his commentaries, that "with us the degree of doctor, in any of the faculties, together with that of sergeant-at-law, has always been held superior to the rank or degree of esquire, and next to the honour of knighthood." La Roque says that at the Council of Basle 1431, the Emperor Sigismund ordered that doctors should precede knights.

* One of the witnesses stated that an action is pending, brought against Mr. Beale by the Apothecaries' Company, as he has not their licensc.

